# Link Protein 1 Is Involved in the Activity-Dependent Modulation of Perineuronal Nets in the Spinal Cord

**DOI:** 10.3390/ijms25084267

**Published:** 2024-04-12

**Authors:** Judith Sánchez-Ventura, Natalia Lago, Clara Penas, Xavier Navarro, Esther Udina

**Affiliations:** Department Cell Biology, Physiology and Immunology, Institute of Neuroscience, Centro de Investigación Biomédica en Red sobre Enfermedades Neurodegenerativas (CIBERNED), Universitat Autònoma de Barcelona, 08193 Bellaterra, Spainnatalia.lago@uab.cat (N.L.); clara.penas@uab.cat (C.P.)

**Keywords:** perineuronal nets, spinal cord injury, link protein 1, activity-dependent therapy, maladaptive plasticity

## Abstract

One of the challenges of the mature nervous system is to maintain the stability of neural networks while providing a degree of plasticity to generate experience-dependent modifications. This plasticity–stability dynamism is regulated by perineuronal nets (PNNs) and is crucial for the proper functioning of the system. Previously, we found a relation between spinal PNNs reduction and maladaptive plasticity after spinal cord injury (SCI), which was attenuated by maintaining PNNs with activity-dependent therapies. Moreover, transgenic mice lacking the cartilage link protein 1 (*Crtl1* KO mice) showed aberrant spinal PNNs and increased spinal plasticity. Therefore, the aim of this study is to evaluate the role of link protein 1 in the activity-dependent modulation of spinal PNNs surrounding motoneurons and its impact on the maladaptive plasticity observed following SCI. We first studied the activity-dependent modulation of spinal PNNs using a voluntary wheel-running protocol. This training protocol increased spinal PNNs in WT mice but did not modify PNN components in *Crtl1* KO mice, suggesting that link protein 1 mediates the activity-dependent modulation of PNNs. Secondly, a thoracic SCI was performed, and functional outcomes were evaluated for 35 days. Interestingly, hyperreflexia and hyperalgesia found at the end of the experiment in WT-injured mice were already present at basal levels in *Crtl1* KO mice and remained unchanged after the injury. These findings demonstrated that link protein 1 plays a dual role in the correct formation and in activity-dependent modulation of PNNs, turning it into an essential element for the proper function of PNN in spinal circuits.

## 1. Introduction

The extracellular matrix (ECM) is a crucial part of the central nervous system (CNS) that provides structural and biochemical support to cells. While most of the ECM is diffuse and fills all the intercellular space, there are also specialized and highly condensed pericellular coats of ECM, named perineuronal nets (PNNs), that just surround some neurons in the CNS [1]. Perineuronal nets are primarily known for their role in synaptic stabilization and plasticity control [2]. However, other roles, such as ionic buffering, neuroprotection, and neural maturation, have recently been attributed [3]. PNNs are mainly composed of hyaluronan, chondroitin sulfate proteoglycans (CSPGs, including aggrecan, versican, neurocan, and brevican), link proteins, and the glycoprotein Tenascin-R [4]. Among all of them, link proteins are key elements since they are crucial for the formation and maintenance of PNN structures [5]. In vitro, the lack of the gene *Crtl1*, which encodes the link protein 1, prevents the formation of PNNs around PNN-bearing cells [6]. In vivo, it generates attenuated PNNs in the visual cortex [5], the deep cerebellar nuclei [7], and aberrant PNNs in the spinal cord, with an altered proportion of their components [8].

Although PNNs’ structure is quite stable, it can be rearranged by the activity of neurons, facilitating or restricting plasticity [9]. Smith et al. demonstrated that this activity-dependent regulation differs depending on the anatomical location; while physical activity reduced cortical PNNs, the same activity increased spinal PNNs [10]. Likewise, a protocol of voluntary running reduced PNNs in the brainstem sensory nuclei but increased spinal PNNs around lumbar motoneurons [11].

Changes in PNN structure can also be observed after traumatic spinal cord injury (SCI) when alterations in the ECM break the plasticity–stability balance of the mature nervous system. Indeed, spared neurons below the injury level increase their plastic capability and create new connections that enhance spontaneous recovery or lead to maladaptive symptoms such as neuropathic pain and spasticity [12]. Activity-dependent therapies are one of the most successful strategies for rewiring and stabilizing synapses in a functional manner and, thus, reducing maladaptive symptoms after SCI [13]. In a recent study, we showed that after a thoracic SCI, there is a reduction in the thickness of PNNs around lumbar motoneurons, and the application of activity-dependent therapies counteracts this reduction, promoting functional recovery [8]. These findings highlight the link between maladaptive plasticity, activity-dependent therapies, and spinal PNN and situate spinal PNNs in the spotlight of the pathophysiology and recovery after SCI.

**Figure 1:**
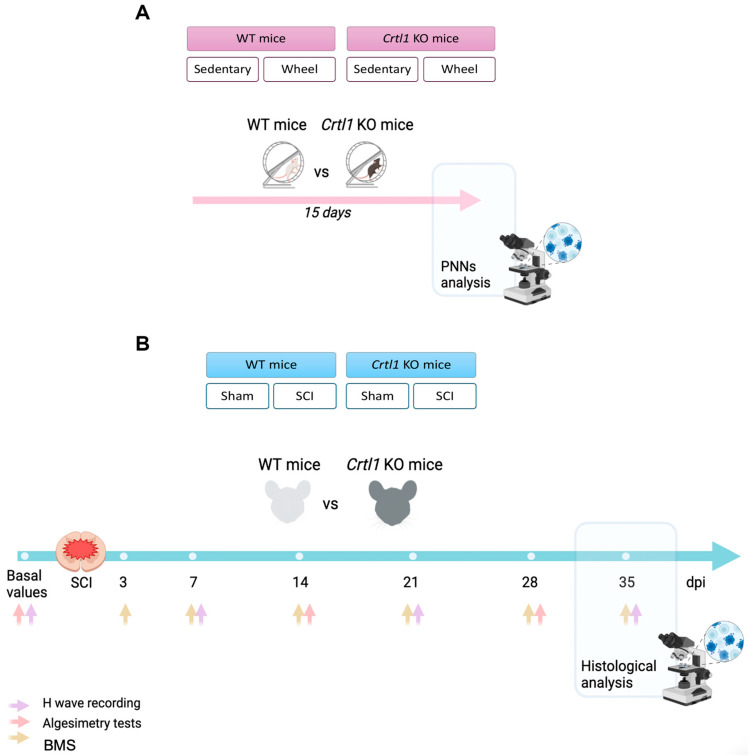
Schematic representation of the experimental design. (**A**). Diagram showing the experimental groups and the timeline of the activity-dependent modulation experiment. (**B**). Schematic diagram of the experimental groups and the timeline of the SCI experiment. Individual images were obtained from Biorender. WT: wild-type; *Crtl1*: cartilage link protein 1; *Crt1*: cartilage link protein 1; KO: knock-out; PNNs: perineuronal nets; SCI: spinal cord injury; DPI: days post-injury.

Thus, the goal of this study was to evaluate the role of link protein 1 in the activity-dependent modulation of PNNs and its importance for the proper function of spinal PNNs. For this purpose, transgenic mice lacking the link protein 1 (*Crtl1* KO) were used. We previously reported that these mice had aberrant spinal PNNs, increased spinal excitability, and presented motor dysfunctions [8]. In the present study, we investigated whether the absence of link protein 1 affects the increase in PNNs observed after physical activity and the decrease in PNNs observed after the inactivity produced by SCI. Thus, functional and histological outcomes produced by a moderate thoracic SCI were compared between *Crtl1* KO and WT mice (Figure 1).

## 2. Results

### 2.1. Link Protein 1 Is Involved in the Activity-Dependent Changes of Perineuronal Nets in the Spinal Cord

**Figure 2:**
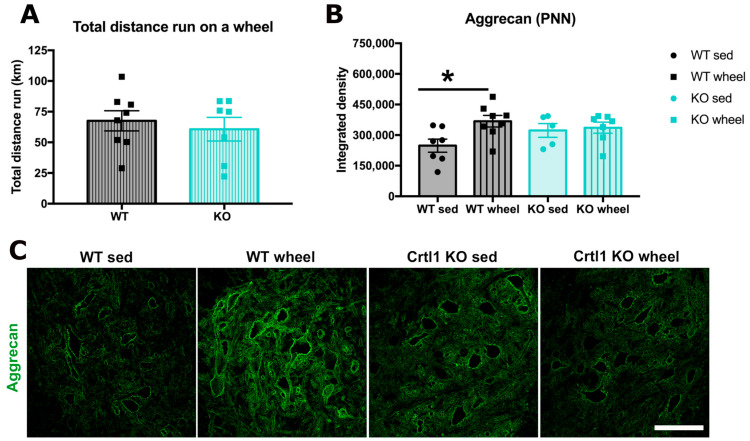
Link protein 1 is implicated in the activity-dependent modulation of PNNs around lumbar MN. (**A**). Total distance run during the 15 days in mice running between 21 and 104 Km total, after excluding extreme runners. (**B**). Quantification of the immunolabeling of the PNN component aggrecan. N_total_ = 27 mice. Bar graphs represent the mean values ± SD. * *p* < 0.05 by one-way ANOVA (F_3,23_ = 3.04, *p* = 0.047) followed by post-hoc test with Bonferroni correction (* *p* = 0.0145). (**C**). Maximal projection of confocal images from the ventral horn of the lumbar spinal cord of WT and *Crtl1* KO mice, showing the expression of aggrecan (green). Scale bar: 100 µm. WT: wild-type; *Crtl1*: cartilage link protein 1; KO: knock-out; sed: sedentary; PNN: perineuronal net.

Our first goal was to determine whether the activity-dependent modulation of PNNs, produced by two weeks of free access to a running wheel, is mediated by the link protein 1. Since we described that *Crtl1* KO mice were hypoactive in a previous paper, we first compared the total distance run between groups. Despite not being significant, KO mice tended to run less distance than WT (44 ± 10 vs. 71 ± 10 km total). Therefore, to ensure that the trend to run less in *Crtl1* KO animals was not influencing our results, we only analyzed animals running a similar range of distances. Thus, we excluded two KO mice that were not running more than 20 km total, and we also excluded an extreme WT runner that ran 114 km total to homogenize the mean distance run between groups (65 ± 9 vs. 53 ± 11 km total in WT and KO mice, respectively, Figure 2A). A significant increase in the integrated intensity of the PNN marker aggrecan was observed in WT animals than run compared to sedentary ones (*p* < 0.05). In *Crtl1* KO mice, voluntary wheel running was not able to modulate aggrecan staining (Figure 2B). Therefore, aggrecan was only significantly increased in WT runners.

### 2.2. Uninjured Crtl1 Knock-Out Mice Mimic the Functional Outcomes Observed in the Injured Wild-Type Mice

**Figure 3:**
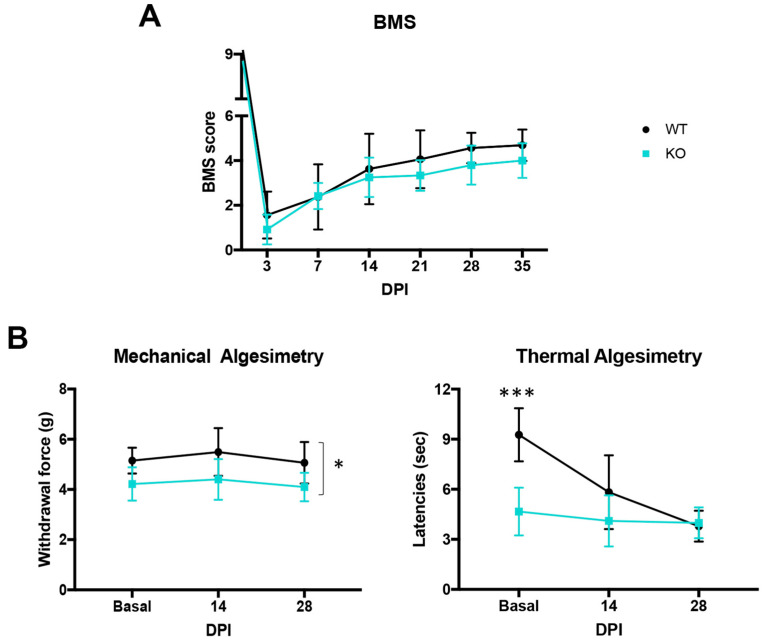
The neuropathic pain produced by the spinal cord contusion was already present in Crtl1 KO mice before the injury. (**A**). Open field locomotion assessed by the BMS scale. (**B**). Neuropathic pain assessment by thermal and mechanical algesimetry tests performed every two weeks. N_total_ = 14 mice. Data represent the mean values ± SD. * *p* < 0.05, *** *p* < 0.001 vs. WT injured mice as calculated by two-way ANOVA (BMS: time F_5,60_ = 74.51, *p* < 0.0001; group F_1,12_ = 1.146, *p* = 0.3; interaction F_5,60_ = 1.273, *p* = 0.28; mechanical algesimetry: time F_2,26_ = 1.031, *p* = 0.37; group F_1,13_ = 18.27, *p* < 0.001; interaction F_2,26_ = 0.049, *p* = 0.95; thermal algesimetry: time F_2,26_ = 13.86, *p* < 0.0001; group F_1,13_ = 28.57, *p* = 0.0001, interaction F_2,26_ = 8.28, *p* = 0.0017) followed by Bonferroni correction for the multiple comparison. BMS: Basso Mouse Scale; DPI: days post-injury; WT: wild-type; KO: knock-out.

The thoracic spinal cord contusion produced similar motor impairment in WT and *Crtl1* KO mice. All injured mice initially presented hindlimb paralysis, followed by a gradual and spontaneous recovery over the next days. No significant differences were found between groups in the BMS scale during follow-up (Figure 3A).

When mechanical and thermal sensitivity were assessed before the injury, we observed a significant decrease in the withdrawal force and in the heating latency in *Crtl1* KO mice compared to WT mice (*p* < 0.05 and *p* < 0.001, respectively) (Figure 3B). These findings suggested that these transgenic mice already presented thermal and mechanical pain sensitivity before the injury. After SCI, WT mice developed thermal hyperalgesia (Figure 3B), which was manifested by a significant decrease in the withdrawal latency over time (*p* < 0.05 basal vs. 14 dpi; *p* < 0.001 basal vs. 28 dpi and 14 vs. 28 dpi *p* > 0.05). However, the same injury did not produce mechanical allodynia in these mice since they maintained the same withdrawal force at 14 and 28 dpi (Figure 3B).

In contrast, *Crtl1* KO mice maintained their withdrawal threshold values, which were already reduced throughout the experiment, suggesting that the injury had no further effect on their pain sensitivity (Figure 3B).

**Figure 4:**
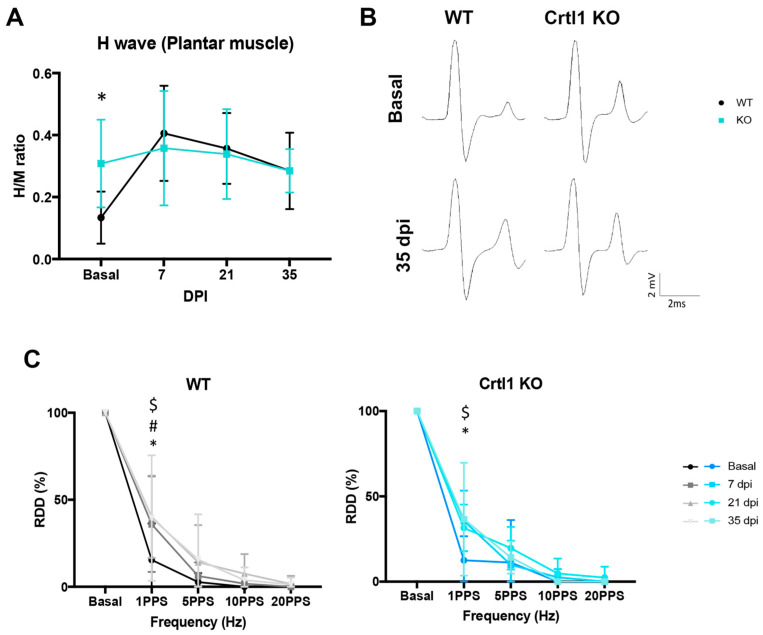
The hyperreflexia produced by the SCI was already observed in Crtl1 KO mice before the injury. (**A**). Quantification of the H_max_/M_max_ changes in the plantar muscle before the injury and at 6, 21, and 35 days after the injury. (**B**). Representative electromyograms showing the initial M wave, resulting from the direct activation of motor axons, and a small wave with longer latency, the H wave, resulting from the monosynaptic activation of lumbar motoneurons by Ia afferents before the injury and at the end of the experiment. (**C**). Depression profile of the H wave after 10 consecutive stimulations at 1, 5, 10, and 20 PPS at the beginning of the experiment, at 6, 21, and 35 days after the injury. N_total_ = 14 mice. Data represent the mean values ± SD. * *p* < 0.05 vs. WT (**A**), * *p* < 0.05 vs. 7 dpi # *p* < 0.05 vs. 21 dpi; $ *p* < 0.05 vs. 35 dpi (**C**) as calculated by two-way ANOVA (H wave recordings: time F_3,39_ = 5.69, *p* = 0.003; group F_1,13_ = 0.38, *p* = 0.551; interaction F_3,39_ = 2.84, *p* = 0.051; *RDD WT:* frequency F_4,28_ = 201.4, *p* < 0.0001; time F_3,21_ = 2.63, *p* = 0.077, interaction F_12,84_ = 1.479, *p* = 0.148; RDD KO: frequency F_4,24_ = 591.7, *p* < 0.0001; time F_3,18_ = 1.39, *p* = 0.28; interaction F_12,72_ = 1.23, *p* = 0.28) followed by Bonferroni correction for multiple comparison. RDD: rate-dependent depression; WT: wild-type; *Crtl1*: cartilage link protein 1; KO: knock-out; DPI: days post-injury; PPS: paired-pulse per second.

Regarding hyperreflexia (Figure 4A,B), the electrophysiological tests performed before the injury showed that *Crtl1* KO mice presented a higher H_max_/M_max_ ratio compared to WT mice (*p* < 0.05). After SCI, *Crtl1* KO mice maintained the same basal levels during the follow-up period. In contrast, 7 and 21 days after the contusion, WT mice presented a significant increase in their H_max_/M_max_ ratio compared to their basal values (*p* < 0.05 basal vs. 7 dpi; *p* < 0.05 basal vs. 21 dpi), reaching the same levels of hyperreflexia than *Crtl1* KO mice at 7, 21 and 35 dpi.

The RDD of the H reflex showed that both groups had almost complete depression of the H wave after 10 consecutive stimuli at 1 Hz (87.5% and 82.5% depression in *Crtl1* KO and WT mice, respectively) before SCI. After SCI, the depression profile changed similarly in both groups, with a significant increase in the % of depression of the H wave at 1 Hz at 7, 21, and 35 dpi in WT (*p* < 0.05) and at 7 and 35 dpi in *Crtl1* KO mice (*p* < 0.05) (Figure 4C).

### 2.3. Lack of Modulation of Perineuronal Nets after Spinal Cord Injury in Crtl1 Knock-Out Mice

**Figure 5:**
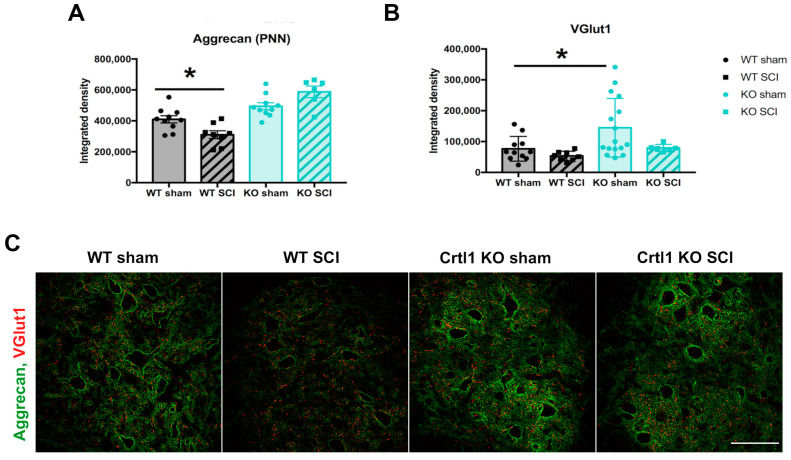
The spinal cord injury reduced lumbar perineuronal nets in WT but not in *Crtl1* KO mice. (**A**). Quantification of PNNs labeled with aggrecan (green) around lumbar motoneurons. (**B**). Quantification of the proprioceptive afferents found in 4µm around lumbar motoneurons, labeled with VGlut1 (red). (**C**). Confocal images representing PNN and the proprioceptive afferents around lumbar motoneurons found in the ventral horn. N_total_ = 34 mice. Scale bar: 100 µm; Bar graphs represent the mean values ± SD. * *p* < 0.05 as calculated by one-way ANOVA (Aggrecan: F_3,30_ = 16.48, *p* < 0.0001, VGlut: F_3,34_= 4.65, *p* = 0.0074) followed by Bonferroni comparison (* *p* = 0.028 for aggrecan and *p* = 0.03 for VGtut1). PNN: perineuronal nets; WT: wild-type; *Crtl1*: cartilage link protein 1; KO: knock-out; SCI: spinal cord injury; VGlut1: vesicular glutamate transporter 1.

Changes in spinal PNNs were studied after SCI in both groups. Aggrecan immunostaining at the lumbar spinal cord revealed that the contusion significantly decreased the thickness of spinal PNNs in injured WT mice compared to the sham group (Figure 5A, *p* < 0.05). In contrast, we did not find differences in the aggrecan immunolabeling of spinal PNNs between sham and injured *Crtl1* KO mice. Then, we studied the proprioceptive inputs to lumbar MNs with the VGlut1 marker (Figure 5B), which is specific for Ia sensory afferents from the muscle spindle [14]. Uninjured *Crtl1* KO mice showed a significant increase in VGlut1 on MNs, whereas the SCI did not modify VGlut1 immunolabeling at 35 days after the injury neither in WT nor in *Crtl1* KO mice.

### 2.4. Lack of Link Protein 1 Modifies Glial Reactivity after Spinal Cord Injury

**Figure 6:**
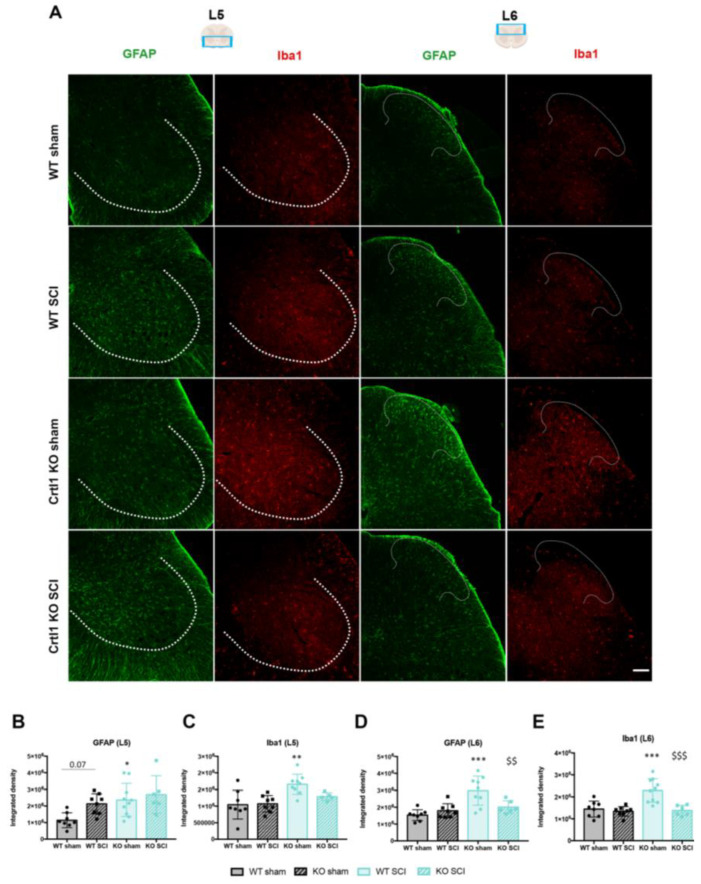
The lack of link protein 1 and the spinal cord injury produces different changes in glial reactivity. (**A**). Representative microphotographs of the ventral (L5 region) and dorsal horn (L6 region) of the lumbar spinal cord in which astrocytes (GFAP; in green) and microglia (Iba1; in red) are shown. Tentative boundaries to define ventral and dorsal horns are labeled by doted lines. (**B**,**C**). Quantification of astrocytes and microglia labeled with GFAP and Iba1 at the ventral horn of L5 spinal cord. (**D**,**E**). Quantification of the intensity of the glial markers GFAP and Iba1 at the dorsal horn of L6 spinal cord. Scale bar: 100 µm; Bar graphs represent the mean values ± SD. * *p* < 0.05, ** *p* < 0.01, *** *p* < 0.001 vs WT sham, ^$$^ *p* < 0.01, ^$$$^ *p* < 0.001 vs KO sham by one-way ANOVA (GFAP L5: F_3,28_ = 4.88, *p* = 0.008; Iba1 L5: F_3,26_ = 7.09, *p* = 0.001; GFAP L6: F_3,27_ = 10.9, *p* < 0.0001; Iba1 L6: F_3,28_ = 12.83, *p* < 0.0001) followed by post-hoc test with Bonferroni correction. WT: wild-type; *Crt1*: cartilage link protein 1; KO: knock-out; SCI: spinal cord injury.

Astroglial and microglial reactivity was studied at the ventral and dorsal horns of the lumbar spinal cord. Before the injury, *Crtl1* KO sham mice showed a significant increase in the intensity of the markers GFAP and Iba1 at the ventral horn (GFAP: *p* < 0.05; Iba1: *p* < 0.01; Figure 6B,C) and the dorsal horn (*p* < 0.001; Figure 6D,E) compared to WT sham mice. The estimated density of microglia was similar in both WT and transgenic mice; therefore, the increased immunoreactivity against Iba1 could be caused by the longer ramifications observed in the basal microglia of *Crtl1* KO mice (Supplementary Figure S1). At 35 dpi, astroglial reactivity in the ventral horn of WT mice tended to increase compared to the sham group but without reaching significance (*p* = 0.07). In contrast, SCI did not modify astroglial reactivity in the *Crtl1* KO-injured mice (Figure 6B). The same finding was observed in microglia reactivity since neither WT nor *Crtl1* KO injured mice showed a significant increase with respect to the respective sham group (Figure 6C).

At the dorsal horn, no differences in both glial markers were found in WT mice, indicating that the inflammatory milieu generated below the injury did not reach the L6 segment or it had already subsided. Remarkably, glial immunoreactivity was higher in *Crtl1* KO sham mice than in WT mice, and unexpectedly, SCI produced a significant reduction in GFAP (Figure 6D) and Iba1 (Figure 6E) intensity compared to the *Crtl1* KO sham group (*p* < 0.01 and *p* < 0.001, respectively).

## 3. Discussion

The developmental formation of PNNs is activity-dependent and coexists with the end of the critical period of plasticity and maturation of the CNS [9,15,16]. Once condensed PNNs are formed, they still present an activity-dependent dynamism that is crucial for CNS function [17]. However, the mechanisms involved in PNN modulation are not well understood. Here, we have demonstrated that link protein 1 participates in the activity-dependent remodeling of spinal PNNs. Our data provide evidence that link protein 1 not only contributes to PNN structure but also participates in the modulation of PNN structure by activity. This dual role is reflected in the following findings: (1) neither activity-dependent therapy nor SCI produces significant changes in spinal PNNs components and thickness in *Crtl1* KO mice, although they respectively increase and reduce spinal PNNs thickness in WT mice; (2) aberrant PNNs in *Crtl1* KO mice produce maladaptive changes in spinal circuits that result in hyperreflexia and hyperalgesia, as observed after SCI in WT mice, a condition that also alters PNN integrity.

### 3.1. Link Protein 1 Mediates the Activity-Dependent Remodeling of Perineuronal Nets

The importance of link protein 1 in the appearance of mature PNN was proved a decade ago in vitro and in vivo [5,6]. Now, we have reported that link protein 1 orchestrates not only the developmental activity-dependent formation of PNNs but also its activity-dependent remodeling in adulthood.

Link proteins are comprised of four members, three of them expressed in the CNS (*Crtl1* or *Hapln1*, *Hapln2,* and *Bral2* or *Hapln4* genes) [18] but just two in PNNs (*Crtl1* and *Bral2* genes) [19]. The latter ones regulate the micro-organization of PNNs by interacting with lecticans and hyaluronan. The *Crtl1* gene allows the capture of aggrecan into the hyaluronan backbone, whereas the *Bral2* links brevican with hyaluronan [20,21]. Although both link proteins are considered organizers of PNNs, link protein 1 triggers the activity-dependent formation of the net since its expression reaches a peak that coincides with the onset of PNNs appearance [5]. Dark-rearing experiments were the first to assess this activity-dependent formation: early sensory deprivation prevents PNN formation and reduces *Crtl1* gene expression in the visual cortex. The subsequent light exposure returned link protein 1 expression and, thus, PNN formation [2,5]. In the present study, PNN remodeling was assessed in the mature spinal cord using transgenic mice lacking the link protein 1 (*Crtl1* KO mice) and submitting them to two different activity-dependent conditions that enhance (locomotor activity) or reduce (SCI) neuronal activity.

#### 3.1.1. Remodeling of Spinal Perineuronal Nets after a Locomotor Activity

Wang et al. were the first to demonstrate that activity increases spinal PNNs [22]. This finding was initially overlooked despite demonstrating that activity modulates spinal and cortical PNN in opposite directions. In fact, it was not until 2015 that the differential activity-dependent modulation of spinal PNNs was highlighted [22,23]

We have observed that WT mice that run on a wheel significantly increase their spinal PNN component aggrecan after the two-week protocol compared to sedentary WT mice. The voluntary wheel protocol was chosen considering previous works that described that an intense duration and intensity were necessary to increase spinal PNNs [11,24]. In contrast, *Crtl1* KO mice that ran on the wheel did not modify aggrecan staining, indicating the importance of link protein 1 on PNN remodeling. However, we have previously shown that transgenic mice are hypoactive and have some motor problems [8], which could limit their capacity to run. Indeed, when comparing the distance run between WT and *Crtl1* KO mice, we observed a non-significant trend in transgenic mice to run less distance than WT mice. These differences were mainly due to extreme runners, and therefore, we decided to exclude them. A histological analysis was performed with animals that run overlapping distances. Thus, we can conclude that link protein 1 is implicated in modulating PNN in the mature nervous system. Foscarin et al. (2011) found similar results in which exposure to an enriched environment significantly decreased the mRNA expression of PNN components in WT mice but not in *Crtl1* KO mice [7].

#### 3.1.2. Remodeling of Spinal Perineuronal Nets after Spinal Cord Injury

Prior studies have already described changes in PNN remodeling following SCI in WT mice [11,25,26]. In agreement with our previous results analyzing the changes in PNN structure after a thoracic cord contusion [11], we observed reduced PNN thickness around disconnected lumbar motoneurons. However, in the present study, PNN reduction was less pronounced, probably due to a faster spontaneous recovery that resulted in more propriospinal inputs on denervated neurons, which eventually offset PNN reduction.

Considering PNNs reduction after SCI, similar findings were detected after a spinal hemisection in goldfish. In particular, it was observed that SCI produced an alteration in the sulfation pattern of CSPGs, specifically a downregulation of the CS-C around MNs [26]. Other works did not report a reduction in lumbar PNNs after a thoracic SCI [27]. This difference could be explained by the use of the marker Wisteria Floribunda lectin (WFA), which only stains 30% of α-MN compared to the 80% stained with aggrecan [28,29]. Thus, these differences could have masked a possible change in PNNs after injury.

In contrast to WT mice, aberrant PNNs in *Crtl1* KO remained unchanged after the spinal cord contusion, suggesting that the lack of the link protein 1 impairs the activity-dependent modulation of PNNs after the injury. The mechanisms implicated in PNNs remodeling around disconnected neurons are probably the same endogenous mechanisms involved in the regular turnover of PNNs. In this process and following the quadripartite synapse concept [30], neurons and glial cells participate in either synthesizing or digesting PNN components through the release of metalloproteases (MMP) or ADAMTs (A Disintegrin and Metalloproteinase with Thrombospondin motifs) [31,32]. After SCI, the equilibrium between synthesis and degradation may be disrupted and tipped toward degradation. In fact, different researchers have described an upregulation of MMPs and ADAMTs after SCI [33,34]. In our study, we did not observe significant changes in the gliosis after the lesion, suggesting that the reduction in lumbar PNNs is not directly caused by microglia digestion but rather by a reduction in the synthesis of PNN components or the release of neural proteinases.

Contrarily, the *Crtl1* KO mice presented increased astrogliosis in both ventral and dorsal horns before the injury. Since during the first postnatal weeks, there is a robust proliferation of astrocytes [35,36], which may allow plasticity at these early stages of development, the astrogliosis observed in *Crtl1* KO mice may contribute to the maintenance of spinal circuits in an immature state in the adulthood, mimicking those in the critical period, before condensed PNNs emerged.

### 3.2. Link Protein 1 Plays a Role in Neural Excitability

Many studies have tried to investigate the functional role of spinal PNNs by applying the enzyme Chondroitinase ABC (chABC), which digests the glycosaminoglycans (GAGs) found in their CSPGs [37,38]. Nevertheless, the chABC application cannot determine the extent to which PNNs contribute to post-SCI maladaptive symptoms, as this enzyme indiscriminately digests both diffuse ECM and PNNs, and only 2% of CSPGS are found in PNNs [39]. Thus, harnessing the strengths of transgenic mice with aberrant PNNs is a good alternative to elucidate the functional implication of PNNs and PNN remodeling in the healthy and injured spinal cord.

In the present study, we have observed that *Crtl1* KO mice presented hyperreflexia and hyperalgesia, mimicking the same phenomena observed in WT mice after SCI. Such maladaptive symptoms remained unchanged after the lesion in the *Crtl1* KO mice, indicating that the SCI did not affect the already disorganized spinal networks of the transgenic mice. Thus, alterations in PNN integrity, either produced by the lack of the link protein 1 or by the reduction in aggrecan after injury, may contribute to disorganized spinal circuits and trigger maladaptive changes. Tansley et al. described that the appearance of neuropathic pain after a peripheral nerve injury was caused by the digestion of spinal PNNs in the dorsal horn [40]. Similarly, the development of excessive pain sensitivity in *Crtl1* KO mice may be caused by alterations of PNNs rather than changes in glial cells. In fact, hyperalgesia was manifested at baseline and persisted throughout the follow-up after SCI, whereas microglial cells were overactivated already before the injury and downregulated afterward. Regarding hyperreflexia, we have previously described increased excitability in monosynaptic and polysynaptic reflex circuits in *Crtl1* KO mice compared to WT mice, which was accompanied by a general increase in excitatory but not inhibitory synapses [8]. Besides a basal increase in VGlut1 synapses around MN in transgenic mice, as we reported again in this paper (Figure 5B), in our previous study, we also described an increase in other excitatory synapses, such as C-boutons, whereas the density of gabaergic inhibitory synapses was similar in *Crtl1*KO and WT mice. Thus, we hypothesize that the spinal disorganization produced by changes in PNN integrity turns those PNN-bearing neurons and their respective circuits into an immature state. This explanation is supported by the similarities between immature neurons without PNN during the critical period, neurons with immature PNN in *Crtl1* KO mice, and reduced PNN thickness in denervated neurons after a SCI. During the critical period, excitatory circuits prevail over inhibitory ones, as the high intracellular Cl- levels favor depolarizing responses. The increase in excitatory synapses was also observed in the spinal cord of *Crtl1* KO mice [8], and the increased intracellular [Cl-] in denervated neurons after a SCI due to KCC2 downregulation [11,41]. In fact, KCC2 cotransporter and PNNs are tightly related since their developmental expression in the spinal cord coincides at P14 [29,42]. In addition, the juvenile levels of plasticity observed in both the visual cortex and the spinal cord of *Crtl1* KO mice [5,8] resemble the boost of plasticity observed in spared neurons after injury [43] and in immature neurons during the refinement of synaptic wiring [44].

When link protein 1 is present, PNN alterations and their functional consequences can be counteracted by the activity-dependent remodeling of PNN orchestrated by this protein. This is observed in the dark/light exposure experiments and in SCI experiments, followed by physical rehabilitation [2,11]. Since the maturation of PNN is activity-dependent, the lack of the link protein 1 might impede the maturation of spinal circuits, which remain immature in adulthood.

Altogether, this work has demonstrated the implication of the link protein 1 and, thus, PNNs in the proper function of the spinal cord. Alterations in this net have a clear impact on the pathophysiology of a SCI.

## 4. Materials and Methods

### 4.1. Experimental Groups

Transgenic mice used in the present work were provided by Dr. Pizzorusso and maintained in the Animal Facility of the Universitat Autonoma de Barcelona (UAB). Their generation and phenotype were described previously [8,45].

Adult (8–10 weeks) female and male mice were used in this study. Two different experiments were carried out to determine the activity-dependent modulation of spinal PNNs of WT and *Crtl1* KO mice (Figure 1). In the first experiment, four groups were generated to evaluate changes in spinal PNNs after exposing mice to a voluntary wheel-running protocol for 15 days. Thus, the groups were WT sedentary (*n* = 7), WT wheel (*n* = 9), *Crtl1* KO sedentary (*n* = 5), and *Crtl1* KO wheel (*n* = 9). For the second experiment, four groups were generated to evaluate the effect of inactivity produced by SCI on PNNs.

Thus, WT and *Crtl1* KO mice were subdivided into WT sham (*n* = 10), WT SCI (*n* = 8), *Crtl1* KO sham (*n* = 10), and *Crtl1* KO SCI (*n* = 6). Experimenters were blinded to genotypes during behavioral and histological evaluations.

The experimental procedures were approved by the Experimental Ethical Committee (CEAAH) of our institution and conducted following the animal welfare guidelines of the European Communities Council Directive 2010/63/EC. Mice were housed in groups at 22 °C (±2 °C), kept on a 12:12 light/dark cycle, and received water and food ad libitum.

### 4.2. Spinal Cord Injury

Mice were anesthetized with an intraperitoneal injection of ketamine (90 mg/kg) and xylazine (10 mg/kg) in a saline solution. Then, a longitudinal incision was made in the skin of the dorsum, the vertebrae were accessed, and laminectomy was performed to expose the dura at the low thoracic level. After laminectomy, a moderate contusion was performed at T11 using the Infinite Horizon Impactor device (50 KDyn, tissue displacement between 300 and 490 μm). After the surgery, animals were kept on a thermostatically regulated heating pad until completely awake and received 1 mL of saline solution subcutaneously to prevent dehydration. Postoperative care consisted of subcutaneous injections of buprenorphine (0.1 mg/kg) during the following three days and bladder expression twice a day until the voiding reflex was re-established. For the sham group, only laminectomy was performed.

### 4.3. Activity-Based Therapy Protocol

WT and *Crtl1* KO mice of the wheel groups were housed in a cage equipped with a free-to-access running wheel (364 × 258 × 350 mm; Activity Wheel Cage System for mice, Tecniplast, Buguggiate, Italy). The wheels were connected to a wheel lap counter to calculate the running distance daily using the diameter of the wheel (21 cm) and a revolution correction. Animals were housed alone to determine the running distance of each animal.

### 4.4. Functional Assessment

#### 4.4.1. Locomotion Assessment

Motor recovery was studied in an open field using the Basso Mouse Scale (BMS), a nine-point scale where 0 means total paralysis and 9 normal locomotion. The test was performed 3, 7, 14, 21, 28, and 35 days post-injury (DPI). The evaluation was performed by two researchers blinded to the mice genotype. The score of each paw was averaged for each animal on each testing day. As an exclusion criterion, those mice that showed a BMS >3 the day after the contusion were excluded from the experiment.

#### 4.4.2. Algesimetry Tests

Thermal and mechanical algesimetry tests were performed preoperatively to determine baseline values and at 14 and 28 days after the injury to assess the appearance of hyperalgesia. Before testing sessions, mice were acclimatized to the room and habituated to the testing chambers for 15 min. Then, tests were performed by a blinded researcher.

Mechanical allodynia was assessed using an electronic Von Frey device (Bioseb, Vitrolles, France). Mice were placed in individual compartments with grid bases, and a Von Frey metal tip was applied to the plantar surface of the hindpaw. An increasing mechanical force was applied until a paw withdrawal response resulted. Thermal hyperalgesia was evaluated with the Plantar Algesimeter device (Ugo Basile, Gemonio VA, Italy). Briefly, a light beam (intensity= 30 mW/cm^2^) was pointed to the hind paw until the animal withdrew the paw. The temperature reached by the response was measured. The cutoff time was defined as 20 s. In both tests, three values were obtained for each paw, and the mean values of both paws were averaged for each animal.

#### 4.4.3. Electrophysiological Tests

Hyperreflexia was evaluated prior to the injury and at 7, 21, and 35 days after the injury with electrophysiological tests. Tests were conducted under general anesthesia with ketamine 90 mg/kg and xylazine 10 mg/kg since it has negligible effects on electrophysiological recordings [46]. During the electrophysiological evaluation, a heating pad was used to maintain the body temperature, and a dissection microscope was used to ensure reproducibility of needle location between mice.

Recorded signals were amplified and visualized on a digital oscilloscope (Tektronix 450S, Beaverton, OR, USA), then fed to a PowerLab 4ST unit, and LabChart 7.3.8 software (AD instruments, Colorado Springs, CO, USA) was used for signal analysis.

Hyperreflexia was studied by measuring the H wave response, the electrical counterpart of the stretch monosynaptic reflex. Specifically, we measured the amplitude ratio of the H wave and its rate-dependent depression (RDD) in the interosseous plantar muscle.

Firstly, the sciatic nerve was stimulated by delivering single electrical pulses of 0.01 ms (Grass S88 stimulator, Quincy, MA, USA) with monopolar needles inserted in the sciatic notch. The compound muscle action potential (CMAP) of the plantar muscle was recorded using microneedles inserted in the belly of the muscle as active electrodes and on the fourth toe as a reference. The maximal baseline to peak amplitude of the M wave (M_max_: direct muscle response) and the maximum amplitude of the H wave (H_max_: monosynaptic reflex) were measured. Then, the H_max_/M_max_ ratio was calculated as an index of excitability of the afferent inputs on spinal efferent motoneurons. M wave values were obtained at supramaximal stimulation, whereas the H_max_ was elicited by progressively increasing the intensity of the electrical stimuli until reaching the maximum amplitude of the H wave.

The RDD of the H wave consists of measuring the changes in the amplitude of the H wave with repetitive stimulations. Alterations in this parameter are a hallmark of hyperreflexia. Trains of ten consecutive pulses, using the stimulation intensity that produced the H_max_, were delivered at different frequencies (1, 5, 10, and 20 Hz), with at least 30 s rest between each train. RDD values were expressed as the percentage of the ratio between the last and the first pulse. Then, a curve expressing the % RDD (Y-axis) at different frequencies (X-axis) was generated.

### 4.5. Histological Evaluation

In the voluntary wheel experiment, mice were euthanized 15 days after the beginning of the experiment, whereas in the SCI experiment, mice were euthanized 35 days after the contusion. In both cases, mice were deeply anesthetized (200 mg/kg of pentobarbital) and were transcardially perfused with cold 4% paraformaldehyde (PFA) in 0.1 M phosphate buffer (PB). Spinal cords were harvested and post-fixed in 4% PFA over 2 h. Then, they were cryopreserved in 30% sucrose solution in PB at 4 °C.

Lumbar spinal cords (L4–L5) were transversally cut on a cryostat (20 µm thick sections) and collected onto gelatine-coated glass slides. L4–L5 sections were used to analyze PNN components (aggrecan), proprioceptive afferents (VGlut1: vesicular glutamate transporter 1), and glial reactivity (astrocytes, stained with GFAP, and microglia stained with Iba1) in the ventral horn. L6 sections were used to evaluate glial reactivity in the dorsal horn. For immunofluorescence staining, sections were permeabilized with phosphate buffer saline Triton 0.3% (PBST 0.3%), and nonspecific interactions were blocked with 10% normal donkey serum for 1 h at room temperature (RT). Then, sections were incubated overnight (ON) at 4 °C with primary antibodies (Table 1). After washes, immunoreactive sites were revealed using species-specific secondary antibodies (1:200; Table 1). After 2 h of incubation at RT, sections were washed and mounted with Fluoromount medium (Southern Biotech, Birmingham, AL, USA).

#### Histological Analysis

For PNN components and glutamatergic synaptic markers, images were taken with a confocal laser-scanning microscope (around 30 steps, z-step size of 0.5 μm, Leica TCS SP5) at 40×. For each mouse, 4 images of the spinal cord were taken in which a minimum of 30 alpha MNs (located within the ventral horn and presenting an area > 350 μm^2^ [47]; Supplementary Figure S2) were measured. PNN and VGlut1 analysis were performed following these steps: the maximal projection of the z-stacks was performed, and then the background was corrected. Afterward, a band of 4 μm around the cell body of alpha MNs was delimited to measure the integrated density of that region. Image analysis was performed by Fiji software (http://imagej.nih.gov/ij/).

For glial reactivity, grayscale microphotographs were captured at 20× in the ventral and dorsal horn. After background correction, the threshold was defined for all the microphotographs of each marker. Then, the integrated density of immunoreactivity was measured in a region of interest (ROI) of 302,141 μm^2^ in the ventral horn and a ROI of 301,692 μm^2^ in the dorsal horn. At least eight sections per marker from each mouse were used to calculate the average value. The estimated density of microglia cells in basal conditions was measured by manually counting the number of Iba1+ cells in a ROI of 1 mm^2^ of the ventral horn in 3 random sections of sham animals from each group. Images were acquired with a digital camera Nikon DS-Ri2 attached to a Nikon Eclipse Ni-E microscope (Stroombaan, The Netherlands). Image analysis was performed by Fiji software.

### 4.6. Statistical Analysis

Statistical analysis was performed using GraphPad Prism 7 software, and data was expressed as mean ± standard deviation (SD). A normal distribution was confirmed using a Shapiro–Wilk test. For the voluntary wheel experiment, the distance run during the 15 days was analyzed with a two-way repeated measures ANOVA, whereas the total distance run was studied with a Student’s *t*-test. Functional tests in the SCI experiment were analyzed by two-way repeated-measures ANOVA with group and time after injury as factors, followed by Bonferroni post-hoc correction. All histological results were analyzed with one-way ANOVA followed by Bonferroni post-hoc correction. Differences were considered significant when *p* < 0.05.

## 5. Conclusions

PNNs’ malleability maintains the nervous system in homeostatic equilibrium. In the present study we have shown that this equilibrium is possible due to the dual role of the link protein 1: it organizes PNN structure and contributes to remodeling PNNs in an activity-dependent manner. Once spinal PNNs are altered, spinal circuits become disorganized, generating maladaptive changes that resemble those observed after SCI. Since activity has the opposite effect on encephalic PNN, further studies should address how this link protein differentially modulates cortical and spinal PNNs.

## Figures and Tables

**Table 1:** List of primary and secondary antibodies.

Primary Antibodies				Secondary Antibodies			
Name	Dilution	Host	Reference	Name	Dilution	Host	Reference
Aggrecan	1:250	Rabbit	AB1031—Millipore (Burlington, MA, USA)	Alexa 488	1:200	Donkey x Rabbit	A21206—Invitrogen (Waltham, MA, USA)
VGlut1	1:300	Guinea Pig	AB5905—Millipore	Cys3	1:200	Donkey x Guinea Pig	706-165-148—Jackson (Scottsdale, AZ, USA)
GFAP	1:1000	Rabbit	AB5804—Millipore	Alexa 488	1:200	Donkey x Rabbit	A21206—Invitrogen
Iba1	1:300	Goat	AB5076—Abcam (Cambridge, UK)	Alexa 594	1:200	Donkey x Goat	A11058—Invitrogen

## Data Availability

The raw data supporting the conclusions of this article will be made available by the authors upon request.

## Supplementary Materials

The following supporting information can be downloaded at: https://www.mdpi.com/article/10.3390/ijms25084267/s1.
