# Supplementary material for: Link Protein 1 Is Involved in the Activity-Dependent Modulation of Perineuronal Nets in the Spinal Cord
Source: Int J Mol Sci. 2024 Apr 12;25(8):4267. doi: 10.3390/ijms25084267 (PMC11050079; doi:10.3390/ijms25084267)
Supplement: Supplementary file 1 [file ijms-25-04267-s001.zip › ijms-2948649-supplementary.pdf]

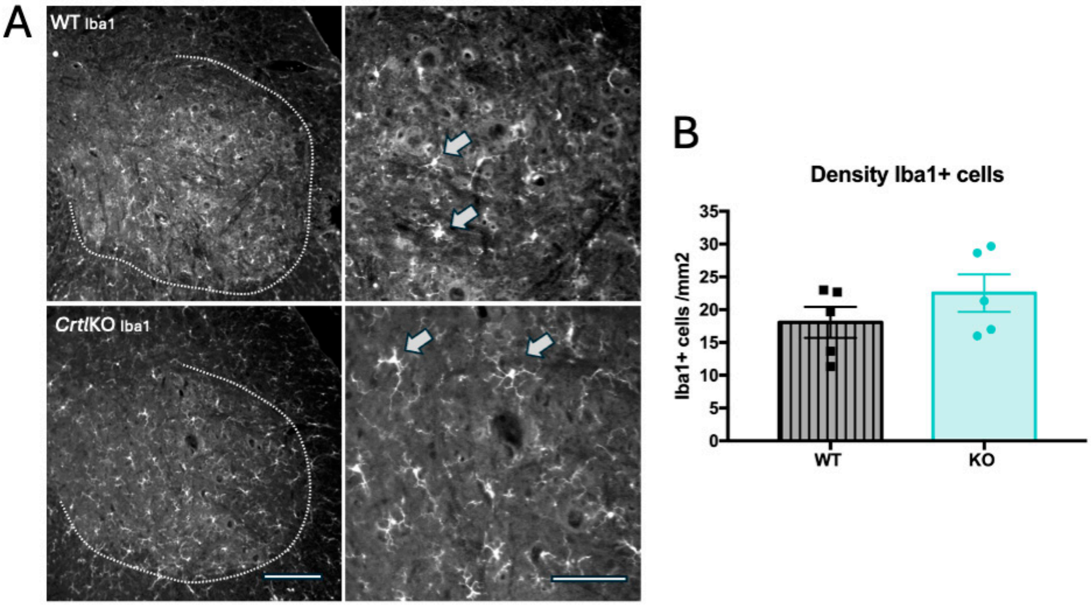

Suppl Fig S1. Similar density of microglia cells in sham WT and *Crtl*KO mice. A) Representative microphotographs of the ventral horn (labeled with intermittent dots) of the lumbar spinal cord stained against microglia (Iba1). A magnification of the central region of the ventral horn is shown in the right panels. Arrows point selected iba1+ cells. In sham *Crtl* KO mice, longer ramifications are observed compared to sham WT. Scale bar: 100 µmB. B) Quantification of the estimated density of Iba1 + in the ventral horn of Sham WT and *Crtl*KO mice.; Bar graphs are representing the mean values ± SD. No significant differences by a t-test.

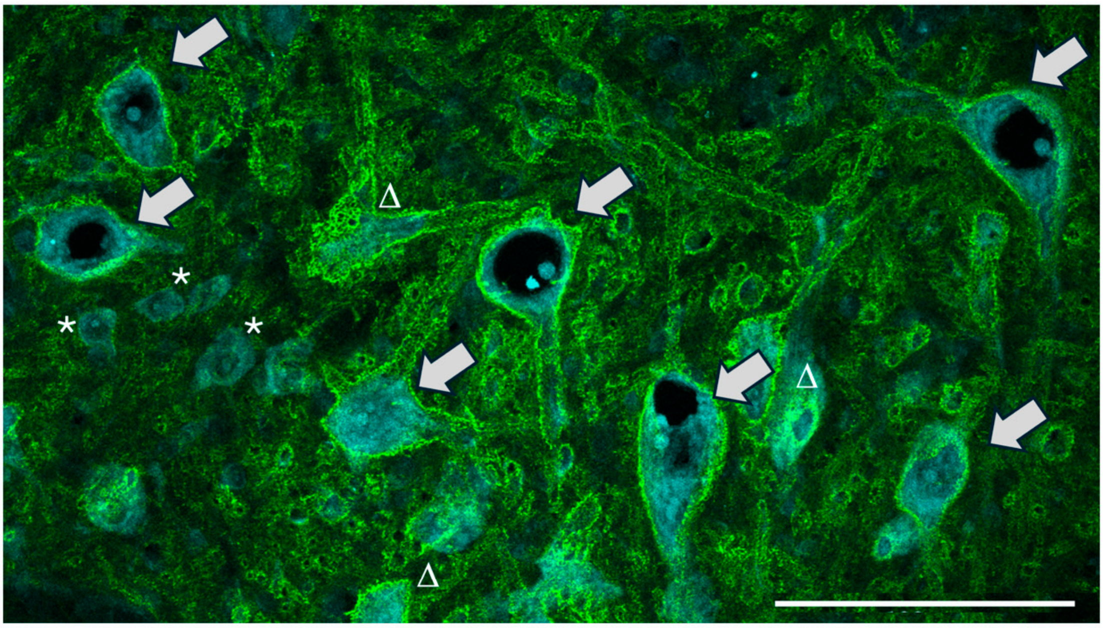

Suppl Fig S2. Maximal projection of confocal images from the ventral horn of the lumbar spinal cord of a WT mice stained with fluoroNissl (blue) and aggrecan (green). Scale bar: 100 µm. Arrows point neurons that were analyzed in this study, where the soma had a triangular shape and size compatible with alpha motoneurons, all surrounded by PNN (green). Δ shows PNN around MN when the section does not include the nucleus. These profiles were not analysed because they did not match the criteria of size and shape compatible with alpha-MN. Smaller neurons (*) that are also present in the ventral horn, not compatible with alpha motoneurons by size (might be gamma MN), lack PNN. Indeed, only alpha MN present PNN in this region of the SC [28].
